# Vanillic Acid Alleviates Acute Myocardial Hypoxia/Reoxygenation Injury by Inhibiting Oxidative Stress

**DOI:** 10.1155/2020/8348035

**Published:** 2020-04-20

**Authors:** Xiuya Yao, Shoufeng Jiao, Mingming Qin, Wenfeng Hu, Bo Yi, Dan Liu

**Affiliations:** ^1^Jiangxi Provincial Key Laboratory of Basic Pharmacology, Nanchang University, School of Pharmaceutical Science, Nanchang 330006, China; ^2^Department of Pharmacy, Changzhou Maternal and Child Health Care Hospital, Changzhou 213000, China; ^3^Department of Pharmacy, The First Affiliated Hospital of Nanchang University, Nanchang 330006, China; ^4^Second Abdominal Surgery Department, Jiangxi Province Tumor Hospital, Nanchang 330029, China

## Abstract

Oxidative stress is an important factor of myocardial hypoxia/reoxygenation (H/R) injury. Our research focuses on how to reduce the cardiac toxicity caused by oxidative stress through natural plant extracts. Vanillic acid (VA) is a phenolic compound found in edible plants and rich in the roots of *Angelica sinensi*s. Experimental studies have provided evidence for this compound's effectiveness in cardiovascular diseases; however, its mechanism is still unclear. In this study, molecular mechanisms related to the protective effects of VA were investigated in H9c2 cells in the context of H/R injury. The results showed that pretreatment with VA significantly increased cell viability and decreased the percentage of apoptotic cells, as well as lactate dehydrogenase and creatine phosphokinase activity, in the supernatant, accompanied by reduced levels of reactive oxygen species and reduced caspase-3 activity. VA pretreatment also restored mitochondrial membrane potentials. Moreover, preincubation with VA significantly attenuated mitochondrial permeability transition pore activity. VA administration upregulated adenosine monophosphate-activated protein kinase *α*2 (AMPK*α*2) protein expression, and interestingly, pretreatment with AMPK*α*2-siRNA lentivirus effectively attenuated the cardioprotective effects of VA in response to H/R injury.

## 1. Introduction

Myocardial hypoxia/reoxygenation (H/R) injury leads to significant morbidity and mortality [1], and oxidative stress is one of the most important factors causing cardiac toxicity. Natural plant extracts have the advantages of few side effects and easy access. Therefore, our research focuses on the use of natural plant extracts to protect the heart against oxidative stress, thereby reducing cardiac toxicity caused by I/R injury. Vanillic acid (VA) is a phenolic compound found in secondary plant products with the molecular formula C_8_H_8_O_4_. VA is widely used in the food industry as flavouring agent, food additive, and preservative. We detected VA in various foods, such as herbs, tea, coffee, wine, and beer [2–7]. VA possesses powerful antioxidant functions, antihypotensive effects, cardioprotective effects, hepatoprotective effects, and antiapoptotic activities [8–11].

Adenosine monophosphate-activated protein kinase (AMPK) is a stress responsive kinase that modulates a number of physiologically and metabolically significant pathways, including apoptosis, energy dynamic balance, and cellular metabolism [12]. AMPK ameliorates cellular antioxidant enzyme systems, such as manganese superoxide dismutase (Mn-SOD) and catalase, consequently reducing oxidant-induced injury [13].

Accordingly, we hypothesized that VA exerts a protective effect against hypoxia/reoxygenation (H/R) injury, which might be related to the AMPK signalling pathway. Therefore, the present study is aimed at addressing the following aims: (1) determine whether VA pretreatment protects H9c2 cells from hypoxia/reoxygenation (H/R) injury and (2) explore the underlying protective mechanisms of VA on hypoxia/reoxygenation (H/R) injury in H9c2 cells.

## 2. Materials and Methods

### 2.1. Reagents

VA (purity: >98%) was purchased from Sigma Chemical Co. (St. Louis, MO, USA). The AMPK*α*2-siRNA lentivirus was purchased from Genechem Co. (Shanghai, China).

### 2.2. Cell Culture

Embryonic rat heart-derived H9c2 cells were purchased from the Chinese Academy of Sciences cell bank. H9c2 cells were incubated at 37°C in a 5% CO_2_ incubator (Forma™ 310, Thermo Fisher, USA) with high glucose (4.5 g/l glucose) DMEM (Solarbio, Beijing, China) by adding 13% FBS (foetal bovine serum, WISENT, Canada) and antibiotics (100 U/ml penicillin and 100 *μ*g/ml streptomycin, Solarbio, Beijing, China). The hypoxia/reoxygenation (H/R) cell model was set up to imitate an ischaemia/reperfusion (I/R) model in vitro [14–17]. Briefly, after different pretreatments, H9c2 cells were cultured in hypoxic solution (sodium lactate 40 mM, NaH_2_PO_4_ 0.9 mM, NaHCO_3_ 6 mM, MgSO_4_ 1.2 mM, HEPES 20 mM, CaCl_2_ 1.8 mM, NaCl 98.5 mM, KCl 10 mM, pH 6.8) and incubated at 37°C with 5% CO_2_ and 0.1% O_2_ in a hypoxic chamber (Proox model C21, BioSpherix Ltd., USA) for 3 h. H9c2 cells were subsequently cultured in reoxygenation solution (glucose 5.5 mM, NaH_2_PO_4_ 0.9 mM, NaHCO_3_ 20 mM, MgSO_4_ 1.2 mM, HEPES 20 mM, CaCl_2_ 1.8 mM, NaCl 129.5 mM, KCl 5 mM, pH 7.4) and incubated at 37°C and 95% O_2_/5% CO_2_ in a reoxygenation chamber for 2 h. All experiments were performed in triplicate.

### 2.3. Experimental Protocol

Cultured H9c2 cells were randomly divided into the following experimental groups:

1. Control group comprised H9c2 cells that were maintained in normoxic conditions with 95% air and 5% CO_2_ in complete medium for 5 h
2. H/R group comprised conditions described in Section 2.2
3. VA+H/R group comprised H9c2 cells that were pretreated with 1.00 mM VA 24 h before H/R treatment
4. VA+NC+H/R group included H9c2 cells that were pretreated with 1.00 mM VA 24 h before H/R treatment and the negative lentivirus 48 h before H/R treatment
5. VA+AMPK*α*2-siRNA+H/R group comprised H9c2 cells that were pretreated with 1.00 mM VA 24 h before H/R treatment and the lentivirus AMPK*α*2-siRNA 48 h before H/R treatment

### 2.4. Cell Viability Assays

The viability of H9c2 cells was determined using the 3-(4,5-dimethylthiazol-2-yl)-5-(3-carboxymethoxyphenyl)-2-(4-sulfophenyl)-2H-tetrazolium (MTS) kit. MTS (Promega, USA) produces a dark blue formazan product when incubated with living cells. H9c2 cells were trypsinized, counted, and seeded in 96-well plates at a density of 1 × 10^4^ cells per well. Following incubation and different treatments, H9c2 cells were treated with 20 *μ*l MTS (5 mg/ml) in 100 *μ*l medium for 2 h at 37°C. After 2 h, the optical density (OD) of each well was determined at a wavelength of 490 nm using a microplate reader (Bio-Rad 680, USA). H9c2 cell viability is indicated as a percentage of controls.

### 2.5. Assessment of LDH and CPK Activities

Lactate dehydrogenase (LDH) and creatine phosphate kinase (CPK) activities were determined using LDH and CPK commercial assay kits (Jiancheng Bioengineering Institute, Nanjing, China) according to the manufacturer's instructions [18]. H9c2 cell supernatants were collected after different treatments, and optical density (OD) was determined at a wavelength of 440 nm and 660 nm to measure LDH and CPK activities, respectively, using a microplate reader (Bio-Rad 680, USA).

### 2.6. Western Blot Analysis

H9c2 cells were washed using cold phosphate-buffered saline (PBS) three times, followed by lysis with Radio Immunoprecipitation Assay (RIPA) and phenylmethanesulfonyl fluoride (PMSF) buffer on ice for 10 min. To remove insoluble material, extracts were centrifuged at 12,000 rpm for 15 min at 4°C. Total protein content was subsequently measured using the Bradford protein assay kit (Beyotime, Shanghai, China). Equal amounts of protein were electrophoresed on 10% sodium dodecyl sulfate polyacrylamide gel electrophoresis (SDS-PAGE) using a gel apparatus (Bio-Rad, USA). Gels were cut according to a prestained, dual color protein molecular weight marker followed by transfer to polyvinylidene fluoride (PVDF) membranes (Solarbio, Beijing, China) which were blocked with 5% nonfat milk in TBST (Tris-Buffer Saline, 0.25% Tween-20) for 2 h. Membranes were incubated with antibodies (1 : 500 dilution) for AMPK*α*2 (Abcam, USA) and *β*-actin (ZSGB-BIO, Beijing, China) overnight at 4°C. Next, membranes were washed nine times for 10 min each in TBST (Tris-Buffer Saline, 0.25% Tween-20). Then, the membranes were incubated with HRP-labelled IgG secondary antibodies for AMPK*α*2 (1 : 5000 dilution) (ZSGB-BIO, Beijing, China) and *β*-actin (1 : 2000 dilution) with shaking at room temperature for 2 h. Next, membranes were washed six times for 10 min each in TBST. To detect the immune complexes, the enhanced chemiluminescence (ECL) method was used. To measure protein expression, densitometry analysis was employed using Image Lab software (Bio-Rad, USA). We used the ratio of the grey value of the target protein and the corresponding beta-actin protein for quantification and statistical analysis.

### 2.7. Determination of Reactive Oxygen Species (ROS) Levels

The fluorescence probe DCFH-DA (2,7-dichlorofluorescein diacetate) is converted to DCFH_2_ which is oxidized to DCF by ROS. DCF emits a green fluorescent signal that can be measured by flow cytometry. This assay was used according to the manufacturer's instructions. H9c2 cells were collected and incubated in a serum-free medium with a final concentration of 10 *μ*M DCFH-DA (KeyGEN BioTECH, Nanjing, China) at 37°C for 30 min in the dark. Fluorescence intensity of dichlorofluorescin (DCF) was measured by flow cytometry (Beckman Coulter, USA) at 488 nm excitation and at 525 nm emission. A.U. is the abbreviation for arbitrary units.

### 2.8. Measurement of Mitochondrial Membrane Potential (ΔΨ_m_)

JC-1 (5,5′,6,6′-tetrachloro-1,1′,3,3′-tetraethylbenzimidazolo carbocyanine iodide) is a dye that changes color from green to red when Δ*ψ*_m_ increases. Mitochondrial membrane potential was measured using JC-1 (KeyGEN BioTECH, Nanjing, China) staining following the manufacturer's instructions. After washing twice with ice-cold PBS, H9c2 cells were harvested and incubated with JC-1 solution at a final concentration of 200 *μ*M at 37°C for 20 min in the dark. Fluorescence was detected by flow cytometry (Beckman Coulter, USA) at excitation and emission wavelengths (ex/em) of 488/630 nm (red) and 488/530 nm (green). Δ*ψ*_m_ was calculated as the red/green fluorescence ratio.

### 2.9. Ca^2+^-Induced Mitochondria Swelling

After different treatments, H9c2 cell mitochondria were extracted using the cell mitochondria isolation kit (KeyGEN BioTECH, Nanjing, China). Next, purified mitochondria were treated with swelling buffer (120 mM KCl, 5 mM KH_2_PO_4_, 20 mM MOPS, and 10 mM Tris-HCl (pH 7.4)). The mPTP open level was assessed using a Ca^2+^-induced mitochondria swelling assay [19]. After adding 200 *μ*M CaCl_2_ to the mitochondria, the opening of the mitochondrial permeability transition pore leads to mitochondrial swelling causing a stable decline in mitochondrial optical density. Due to the ability of the mitochondria to dilate, optical density (OD) was recorded using a microplate reader at 520 nm (Bio-Rad 680, USA). Mitochondrial swelling was recorded as the optical density (OD_1_) at 520 nm, and a second optical density (OD_2_) was recorded 20 min after induction. The optical density at 520 nm was continuously recorded over 20 min. mPTP activity was calculated as ΔOD(OD_1_–OD_2_)/min × 1000.

### 2.10. Measurement of Caspase-3 Activity

Caspase-3 activity was measured using a caspase-3 activity assay kit (Beyotime, Shanghai, China) following the manufacturer's instructions. H9c2 cells were lysed in lysis buffer on ice for 15 min and then collected and centrifuged at 16,000g for 15 min at 4°C to obtain the supernatant. Protein concentration was determined using the bicinchoninic acid (BCA) protein assay kit (Beyotime, Shanghai, China). After adding detection buffer and Ac-DEVD-рNA and incubating at 37°C for 18 h, optical density was detected at 405 nm. Caspase-3 activity was measured using a microplate reader (Bio-Rad 680, USA). A.U. is the abbreviation for arbitrary units.

### 2.11. Analysis of Apoptosis by Flow Cytometry

We previously published methods for measuring apoptosis [20]. Briefly, apoptosis was assessed by flow cytometry using an Annexin V-FITC/PI apoptosis kit (KeyGEN BioTECH, Nanjing, China) following the manufacturer's instructions. Briefly, H9c2 cells were collected and washed three times with ice-cold PBS. Next, collected H9c2 cells were resuspended in 1x Binding Buffer at a final concentration of 5 × 10^5^ cells/ml. After the addition of 5 *μ*l Annexin V-FITC and 5 *μ*l PI, H9c2 cells were incubated in the dark for 15 min at room temperature. Cellular fluorescence was analysed by flow cytometry (ex 488 nm; em 530 nm, Beckman Coulter, USA).

### 2.12. Assessment of Apoptosis by TUNEL Staining

Apoptotic H9c2 cells were analysed by optical microscopy using the terminal deoxynucleotidyl transferase-mediated nick end labelling (TUNEL) staining method, which was performed using a TUNEL Apoptosis Detection kit (Promega, USA) [21]. Apoptotic H9c2 cells are stained brown, identifying them as TUNEL positive. Then, apoptotic H9c2 cells were subsequently observed on a microscope (Olympus, Japan). The number of TUNEL-positive H9c2 cells/the total number of H9c2 cells represents the apoptotic index.

### 2.13. Statistical Analysis

All values are presented as the mean ± SEM. ANOVA was applied to measure the significance of examined data across different groups, followed by *post hoc* analysis to determine individual differences. *p* values ≤ 0.05 were considered statistically significant.

## 3. Results

### 3.1. Pretreatment with VA Ameliorates the Viability of H9c2 Cells and Reduces Levels of LDH and CPK in H9c2 Cells after H/R

**Figure 1:**
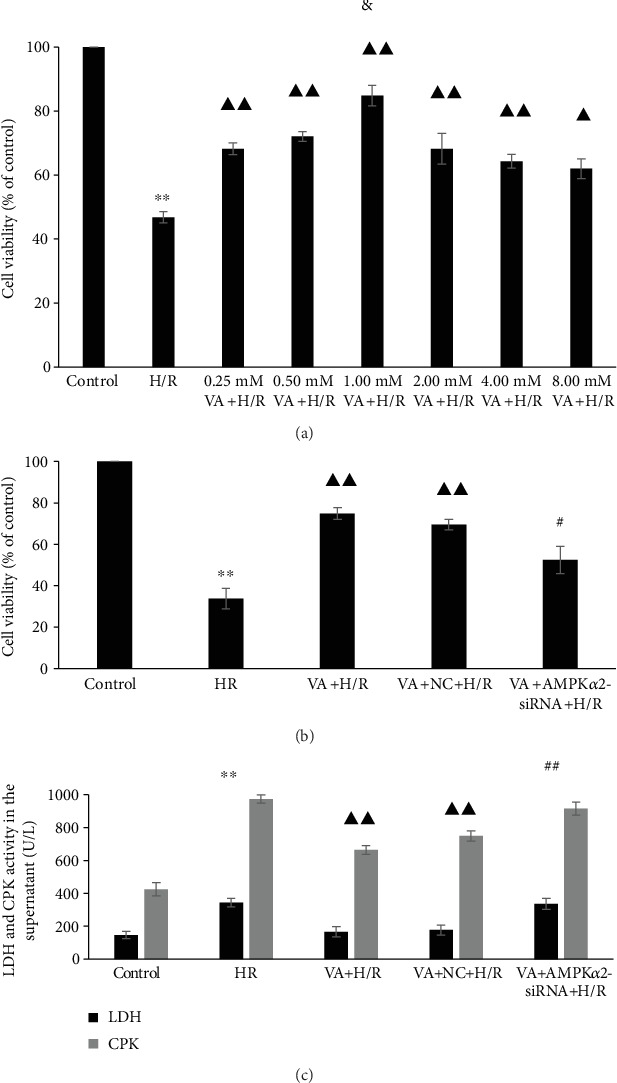
Effects of vanillic acid (VA) on the activities of creatine phosphate kinase (CPK) and lactate dehydrogenase (LDH) activities in the supernatant and cell viability in H9c2 cells subjected to hypoxia/reoxygenation (H/R) injury. (a) ^∗∗^*p* < 0.01 vs. control group; ^▲▲^*p* < 0.01 vs. H/R group; ^▲^*p* < 0.05 vs. H/R group; ^&^*p* < 0.05 vs. 0.50 mM VA+H/R group. (b) ^∗∗^*p* < 0.01 vs. control group; ^▲▲^*p* < 0.01 vs. H/R group; ^#^*p* < 0.05 vs. VA+H/R group. Data are expressed as the mean ± SEM, *n* = 3. M: mol/l. (c) ^∗∗^*p* < 0.01 vs. control group; ^▲▲^*p* < 0.01 vs. H/R group; ^##^*p* < 0.01 vs. VA+H/R group. Data are expressed as the mean ± SEM, *n* = 3.

To measure the effects of VA on H9c2 cells undergoing hypoxia/reoxygenation (H/R), MTS assay was performed on H9c2 cells that were pretreated with different concentrations of VA 24 h prior to H/R. H9c2 cell viability decreased markedly in response to H/R (Figure 1(a), *p* < 0.01 vs. control group). We demonstrated that VA significantly increased H9c2 cell viability. Pretreatment with different concentrations of VA progressively increased viability of H9c2 cell in a concentration-dependent manner. H9c2 cell viability peaked at 1.00 mM VA (*p* < 0.05 vs. 0.50 mM VA+H/R group). However, H9c2 cells pretreated with concentrations of VA higher than 2.00 mM exhibited a significant reduction in cell viability, illustrating that higher VA concentrations cause toxicity. Thus, 1.00 mM VA was selected as the optimal pretreatment concentration for subsequent experiments. In addition, in Figure 1(b), our results show that AMPK*α*2-siRNA caused significantly reduced cell viability (*p* < 0.05 vs. VA+H/R group). Thus, we hypothesized that VA protects H9c2 cells from H/R injury through the AMPK signalling pathway.

To measure injury to H9c2 cell membranes in response to H/R, we evaluated levels of LDH and CPK in the supernatant after H/R treatment. The levels of both LDH and CPK were increased in the H/R group (*p* < 0.01 vs. control group) and markedly decreased in the VA+H/R group (*p* < 0.01 vs. H/R group). Furthermore, the VA+AMPK*α*2-siRNA+H/R group significantly increased the levels of LDH and CPK (*p* < 0.01 vs. VA+H/R group) (Figure 1(c)).

### 3.2. Pretreatment with VA Upregulates AMPK*α*2 Protein Levels in H9c2 Cells Undergoing H/R

**Figure 2:**
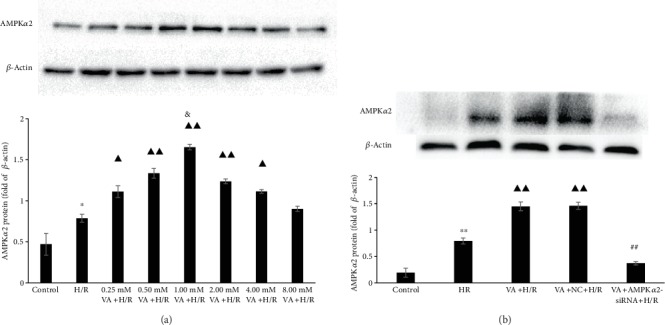
The effect of vanillic acid (VA) on the expression of AMPK*α*2 in H9c2 cells exposed to hypoxia/reoxygenation (H/R) injury. AMPK*α*2 expression was evaluated by western blot, and *β*-actin was used as an internal control. (a) ^∗^*p* < 0.05 vs. control group; ^▲▲^*p* < 0.01 vs. H/R group; ^▲^*p* < 0.05 vs. H/R group; ^&^*p* < 0.05 vs. 0.50 mM VA+H/R group. (b) ^∗∗^*p* < 0.01 vs. control group; ^▲▲^*p* < 0.01 vs. H/R group; ^##^*p* < 0.01 vs. VA+H/R group. Data are expressed as the mean ± SEM, *n* = 3. M: mol/l.

To verify whether the AMPK*α*2 protein was associated with observed cardioprotective activity and optimal pretreatment concentration of VA, we assessed protein levels of AMPK*α*2 by western blot. We pretreated H9c2 cells with different concentrations (0.25, 0.50, 1.00, 2.00, 4.00, and 8.00 mM) of VA for 24 h before H/R. In Figure 2(a), most of VA-pretreated groups exhibited upregulated levels of AMPK*α*2 protein relative to those of control and H/R groups, and the 1.00 mM VA+H/R group exhibited the highest levels of AMPK*α*2 protein (*p* < 0.05 vs. 0.50 mM VA+H/R group). The optimal pretreatment concentration was consistent with that observed by MTS. However, levels of AMPK*α*2 protein were markedly decreased in the VA+AMPK*α*2-siRNA+H/R group relative to those in the VA+H/R group (*p* < 0.01) (Figure 2(b)). Thus, our data indicate that VA exhibits cardioprotective effects against H/R by upregulating AMPK*α*2 protein levels.

### 3.3. Pretreatment with VA Reduces ROS Generation Induced by H/R

**Figure 3:**
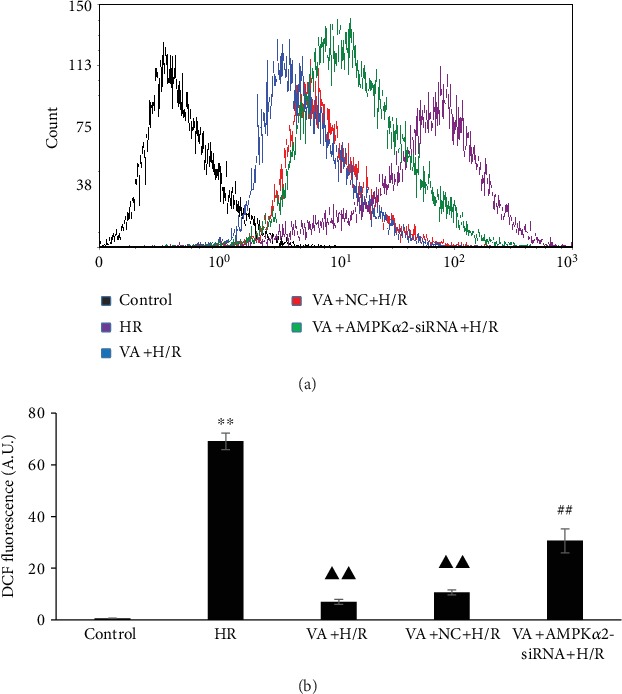
Vanillic acid (VA) pretreatment decreases ROS production in H9c2 cells exposed to hypoxia/reoxygenation (H/R). (a) Flow cytometry analysis of DCF fluorescence. (b) Column bar graph of cell fluorescence for DCF. Data are expressed as the mean ± SEM, *n* = 3. ^∗∗^*p* < 0.01 vs. control group; ^▲▲^*p* < 0.01 vs. H/R group; ^##^*p* < 0.01 vs. VA+H/R group. A.U. is the abbreviation for arbitrary unit.

Relevant experiments were performed to evaluate ROS levels using the DCFH-DA fluorescence probe. ROS levels increased significantly in the H/R group (*p* < 0.01 vs. control group), whereas there was a striking decrease in ROS levels in the VA+H/R and VA+NC+H/R groups (*p* < 0.01 vs. H/R group). As expected, ROS levels markedly increased in the VA+AMPK*α*2-siRNA+H/R group (*p* < 0.01 vs. VA+H/R group and VA+NC+H/R group). These results confirmed that VA reduces ROS generation associated with the AMPK signalling pathway (Figure 3).

### 3.4. Pretreatment with VA Restores Δ*ψ*_m_ in H9c2 Cells Exposed to H/R

**Figure 4:**
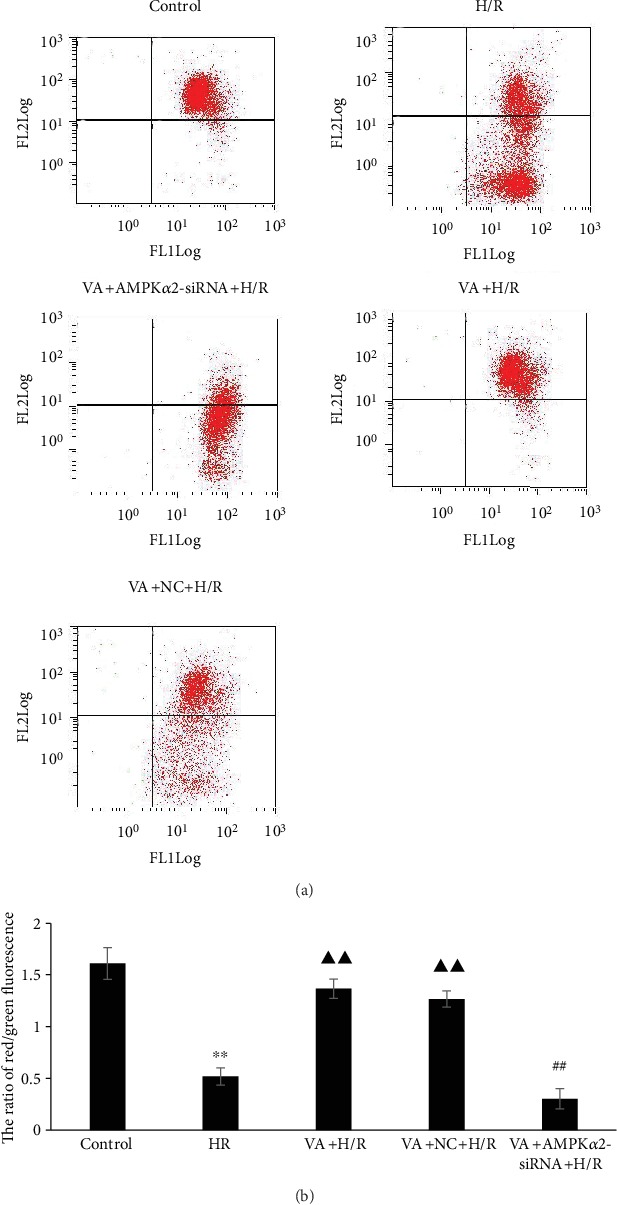
Vanillic acid (VA) pretreatment alleviates loss of ΔΨ_m_ in H9c2 cells exposed to hypoxia/reoxygenation (H/R), while AMPK*α*2-siRNA abrogates this effect. (a) Representative dot plots of flow cytometry. (b) ΔΨ_m_ was calculated with the ratio of red/green fluorescence obtained by flow cytometry. The ratio of fluorescence in the upper right quadrant and lower right quadrant was used to evaluate levels of ΔΨ_m_. Data are expressed as the mean ± SEM, *n* = 3. ^∗∗^*p* < 0.01 vs. control group; ^▲▲^*p* < 0.01 vs. H/R group; ^##^*p* < 0.01 vs. VA+H/R group. JC-1 FL1: JC-1 fluorescence channel 1; JC-1 FL2: JC-1 fluorescence channel 2.

To determine Δ*ψ*_m_ in H9c2 cells after H/R injury, a JC-1 fluorescent probe assay was performed. We used the fluorescence ratio of the upper right quadrant and lower right quadrant to verify Δ*ψ*_m_. The results demonstrated that Δ*ψ*_m_ decreased in response to H/R (*p* < 0.01 vs. control group) (Figure 4). However, pretreatment with VA markedly reduced the loss of Δ*ψ*_m_ in the VA+H/R group (*p* < 0.01 vs. H/R group). In contrast, a disruption in Δ*ψ*_m_ was observed when the AMPK*α*2 gene was knocked down (*p* < 0.01 vs. VA+H/R group), demonstrating that AMPK*α*2-siRNA effectively abrogates the cardioprotective effects of VA against H/R injury.

### 3.5. Pretreatment with VA Prevents Opening of the mPTP Induced by H/R

**Figure 5:**
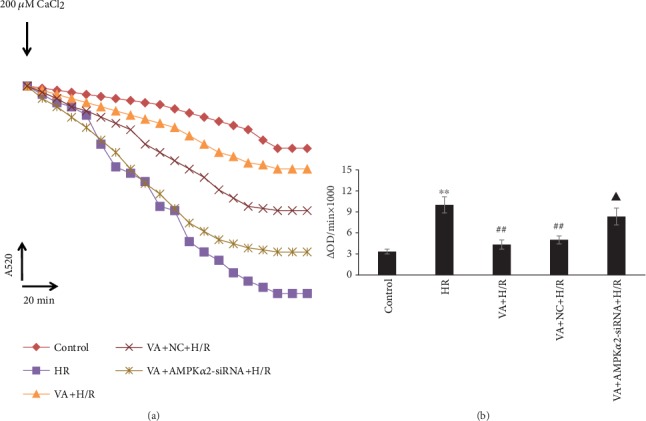
Vanillic acid (VA) preconditioning limited mPTP opening in response to hypoxia/reoxygenation (H/R) in H9c2 cells, while AMPK*α*2-siRNA attenuates this effect. (a) After the addition of 200 *μ*M CaCl_2_, the absorbance value at A520 was monitored over 20 min to reflect the opening of mitochondrial permeability transition pore (mPTP). (b) Changes in absorbance values at 520 nm/min (*Δ*ODmin^−1^) were used to express the extent of mPTP opening (ΔOD = A520_0 min_ − A520_20 min_). Data are expressed as the mean ± SEM, *n* = 3. ^∗∗^*p* < 0.01 vs. control group; ^##^*p* < 0.01 vs. H/R group; ^▲^*p* < 0.05 vs. VA+H/R group.

Ca^2+^-induced mitochondria swelling was used to examine the effects of VA on mPTP opening. We determined the degree of mPTP opening by recording varied optical density values at 520 nm/min (OD/min) over a period of time. As shown in Figure 5, there was a significant increase in *Δ*OD/min in the H/R group (*p* < 0.01 vs. control group); however, pretreatment of H9c2 cells with VA delayed mPTP opening (*p* < 0.01 vs. H/R group). AMPK*α*2 gene knockdown using AMPK*α*2-siRNA lentivirus abolished the cardioprotective effects of VA against H/R injury with respect to inhibiting mPTP opening (*p* < 0.05 vs. VA+H/R group).

### 3.6. Pretreatment with VA Attenuates Caspase-3 Activity in response to H/R

**Figure 6:**
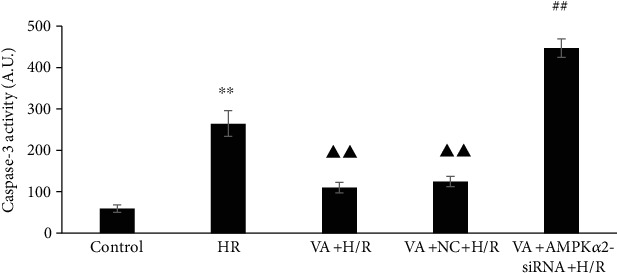
Vanillic acid (VA) pretreatment influences the activity of caspase-3 induced by hypoxia/reoxygenation (H/R) injury, while AMPK*α*2-siRNA abrogates this effect. The column bar graph represents the activity of caspase-3 in the different groups. Data are expressed as the mean ± SEM, *n* = 3. ^∗∗^*p* < 0.01 vs. control group; ^▲▲^*p* < 0.01 vs. H/R group; ^##^*p* < 0.01 vs. VA+H/R group. A.U. is the abbreviation for absorbance unit.

To examine the cardioprotective effects of VA on H/R-induced injury, levels of caspase-3 activity were estimated using a colorimetric method.  Figure 6 shows a significant increase in caspase-3 activity in the H/R group (*p* < 0.01 vs. control group), yet VA pretreatment strikingly decreased caspase-3 activity (*p* < 0.01 vs. H/R group). Furthermore, in line with previous results, a significant increase in caspase-3 activity was observed in the VA+AMPK*α*2-siRNA+H/R group (*p* < 0.01 vs. VA+H/R group).

### 3.7. Pretreatment with VA Decreases H9c2 Cell Apoptosis in response to H/R

**Figure 7:**
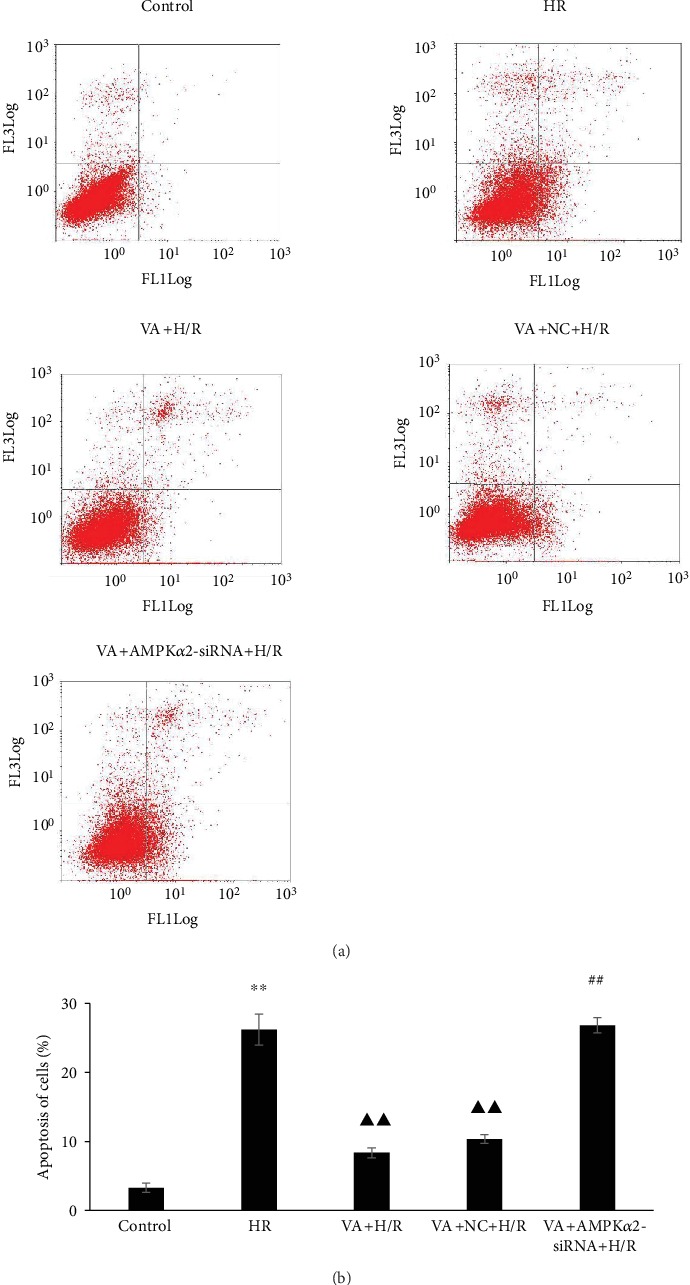
Vanillic acid (VA) pretreatment inhibits apoptosis in H9c2 cells exposed to hypoxia/reoxygenation (H/R), while AMPK*α*2-siRNA abrogates this effect. (a) Representative dot plots of flow cytometry (*x*-axis and *y*-axis represent Annexin V and PI staining, respectively). (b) Evaluation of apoptotic cell populations. Data are expressed as the mean ± SEM, *n* = 3. ^∗∗^*p* < 0.01 vs. control group; ^▲▲^*p* < 0.01 vs. H/R group; ^##^*p* < 0.01 vs. VA+H/R group.

Apoptosis was assessed by flow cytometry using an Annexin V-FITC/PI apoptosis kit. As shown in Figure 7, H9c2 cell apoptotic rates increased markedly in the H/R group (*p* < 0.01 vs. control group), while progressively decreasing in the VA+H/R group (*p* < 0.01 vs. H/R group). Moreover, H9c2 cell apoptotic rates increased again in the VA+AMPK*α*2-siRNA+H/R group (*p* < 0.01 vs. VA+H/R group).

**Figure 8:**
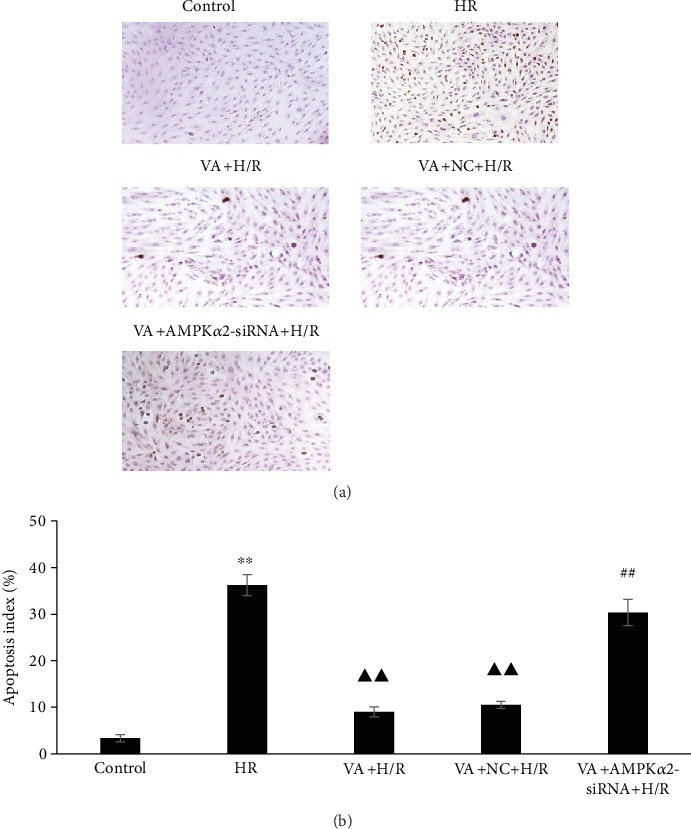
Vanillic acid (VA) pretreatment inhibits apoptosis in H9c2 cells exposed to hypoxia/reoxygenation (H/R), while AMPK*α*2-siRNA abrogates this effect. (a) H9c2 cells were sectioned and analysed for apoptosis using TUNEL staining. The panels show representative histological images. (b) The number of apoptotic cells evaluated by TUNEL is expressed as a percentage. Data are expressed as the mean ± SEM, *n* = 3. ^∗∗^*p* < 0.01 vs. control group; ^▲▲^*p* < 0.01 vs. H/R group; ^##^*p* < 0.01 vs. VA+H/R group.

To further confirm the VA-induced cardioprotective effect against H/R injury, we scored the number of TUNEL-positive H9c2 cells by optical microscopy. As shown in Figure 8, H/R caused a significant increase in the number of TUNEL-positive H9c2 cells in the H/R group (*p* < 0.01 vs. control group). In contrast, the pretreatment of H9c2 cells with VA resulted in significantly reduced TUNEL-positive H9c2 cells (*p* < 0.01 vs. H/R group). In agreement with previous results, the cardioprotective effects of VA were abrogated after AMPK*α*2 gene knockdown using the AMPK*α*2-siRNA lentivirus.

## 4. Discussion

Vanillic acid (VA) is a phenolic compound in edible plants that is enriched in the roots of *Angelica sinensis*. VA exhibits powerful antioxidant functions and possesses cardioprotective, antihypotensive, antiapoptotic, and hepatoprotective activities [8–11]. Experimental studies have provided evidence of this compound's efficacy in cardiac toxicity.

In this study, we found that H9c2 cells pretreated with 1.00 mM VA 24 h prior to H/R significantly increased the viability of H9c2 cells and reduced the levels of LDH and CPK. And our data further confirmed VA pretreatment could inhibit cardiomyocyte apoptosis and protect them against H/R injury.

The cardioprotective effect of VA was extensively investigated, and the potential mechanisms might be associated with countering oxidative stress caused by energy metabolism imbalance, but the mechanism remains to be fully elucidated. AMPK plays an important role in the balance of energy dynamics and is implicated in various diseases [22]. Studies including those from our group have shown that AMPK may be an oxidative stress sensor and redox regulator in addition to its traditional role as an energy sensor and regulator [23, 24]. Moreover, AMPK activation is believed to enhance cellular antioxidant capacity [25, 26]. These findings all led us to hypothesize that VA protects H9c2 cells against H/R injury through antioxidant stress mediated by the AMPK signalling pathway. Therefore, we attempted to detect the expression level of AMPK*α*2 in the treatment of H9c2 anti-H/R injury with different concentrations of vanillic acid and found that the expression level trend of AMPK*α*2 was consistent with the concentration trend of vanillic acid against H/R injury, as shown in Figures 1(a) and 2(a). H9c2 cells pretreated with 1.00 mM VA 24 h before H/R exhibited the highest AMPK*α*2 protein levels. AMPK*α*2 is known to be sensitive to hypoxia and can be activated by increases in AMP. Once H9c2 cells are subjected to H/R damage, they inevitably increase the expression of the AMPK*α*2 protein as an emergency response. However, the increase in AMPK*α*2 protein levels induced by H/R damage is very limited and does not have a protective effect. Our results showed that vanillic acid induces H9c2 cells to produce large quantities of AMPK*α*2 protein and plays a protective role against H/R injury (Figures 1 and 2). So in the following studies, we focused on whether the protective effect of VA against H/R injury was associated with AMPK*α*2.

To verify whether the AMPK*α*2 protein was associated with the cardioprotective activity induced by vanillic acid, we used knockdown of AMPK*α*2 by its specific siRNA. In the recent years, a number of siRNAs have been successfully used in experimental models [27–29]. We specifically suppressed AMPK*α*2 expression using RNA interference, which selectively silenced gene expression by delivering double-stranded RNA molecules into cells, and western blot analysis showed that the expression of target protein AMPK*α*2 could be reduced, as shown in Figure 2(b). Once the expression of AMPK*α*2 was inhibited, the protective effect of vanillic acid pretreatment against H/R injury in H9c2 cells was eliminated. These findings confirmed that VA-induced cardioprotective effects on H/R are associated with the AMPK*α*2 signalling pathway.

Electron transport chain injury results in the production of mitochondrial ROS and oxidative stress damage due to anoxia/reoxygenation [30]. ROS bursts lead to mPTP opening and trigger cellular energetic signals in response to ATP depletion and alterations in ion dynamic balance, ultimately resulting in plasma membrane breach and cell death [31, 32]. It has been demonstrated that mPTP is the central factor in different preconditioning protective mechanisms, and mPTP opening is thought to be important in the interim reperfusion injury [33]. H9c2 cells exposed to H/R injury demonstrated the increased production of ROS, the opening of the mPTP, and the loss of ΔΨ_m_. In contrast, pretreatment with VA attenuated ROS production, the opening of the mPTP, and the loss of ΔΨ_m_.

Oxidative stress damage results in a cellular apoptotic signalling cascade [34]. Caspase-3 is one of the key effectors in this mitochondria-mediated apoptosis pathway. The results showed increased caspase-3 activity in response to H/R injury. In contrast, pretreatment with VA attenuated this effect. Furthermore, pretreatment with VA markedly decreased apoptosis in response to H/R injury in H9c2 cells. To further verify the protective effect of VA against H/R-induced injury, the apoptosis was detected using two methods. The apoptosis results of flow cytometry and TUNEL were consistent, indicating that VA significantly protects H9c2 cells exposed to H/R. It is noteworthy that the protective effect of VA disappeared in response to AMPK*α*2 downregulation.

Our study has proven that VA protects H9c2 cells against H/R-induced injury via reducing the generation of ROS, stabilizing mitochondrial membrane potential, limiting the opening of mPTP, decreasing caspase-3 activity, and ultimately inhibiting cardiomyocyte apoptosis. Furthermore, all protective effects were abrogated by the knockdown of AMPK*α*2-siRNA. Therefore, our findings confirm that VA-induced cardioprotective effects on H/R injury are associated with the AMPK*α*2 protein.

## Data Availability

Interested readers can reproduce our results by using our algorithm.
