# Corrigendum to “Vanillic Acid Alleviates Acute Myocardial Hypoxia/Reoxygenation Injury by Inhibiting Oxidative Stress”

**DOI:** 10.1155/2022/9891489

**Published:** 2022-01-27

**Authors:** Xiuya Yao, Shoufeng Jiao, Mingming Qin, Wenfeng Hu, Bo Yi, Dan Liu

**Affiliations:** ^1^Jiangxi Provincial Key Laboratory of Basic Pharmacology, Nanchang University, School of Pharmaceutical Science, Nanchang 330006, China; ^2^Department of Pharmacy, Changzhou Maternal and Child Health Care Hospital, Changzhou 213000, China; ^3^Department of Pharmacy, The First Affiliated Hospital of Nanchang University, Nanchang 330006, China; ^4^Second Abdominal Surgery Department, Jiangxi Province Tumor Hospital, Nanchang 330029, China

In the article titled “Vanillic Acid Alleviates Acute Myocardial Hypoxia/Reoxygenation Injury by Inhibiting Oxidative Stress” [1], there was an error in Figure 8, where the VA+NC+H/R panel was duplicated as the VA+H/R panel.

The corrected figure with the correct VA+H/R panel is shown in Figure 8.

## Figures and Tables

**Figure 1:**
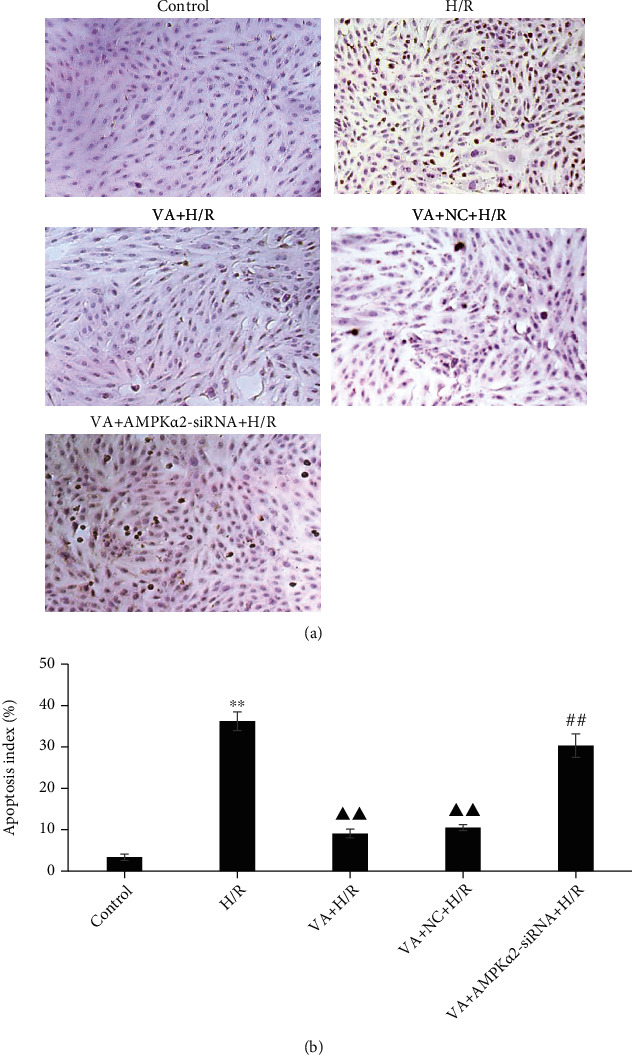
Vanillic acid (VA) pretreatment inhibits apoptosis in H9c2 cells exposed to hypoxia/reoxygenation (H/R), while AMPK*α*2-siRNA abrogates this effect. (a) H9c2 cells were sectioned and analysed for apoptosis using TUNEL staining. The panels show representative histological images. (b) The number of apoptotic cells evaluated by TUNEL is expressed as a percentage. Data are expressed as the mean ± SEM, *n* = 3. ^∗∗^*p* < 0.01 vs. control group; ^▲▲^*p* < 0.01 vs. H/R group; ^##^*p* < 0.01 vs. VA+H/R group.
